# From Moderately Severe to Severe Hypertriglyceridemia Induced Acute Pancreatitis: Circulating MiRNAs Play Role as Potential Biomarkers

**DOI:** 10.1371/journal.pone.0111058

**Published:** 2014-11-03

**Authors:** Fangmei An, Qiang Zhan, Min Xia, Lisha Jiang, Guoming Lu, Mindan Huang, Jizhong Guo, Side Liu

**Affiliations:** 1 Department of Gastroenterology, Wuxi People's Hospital Affiliated to Nanjing Medical University, Wuxi, Jiangsu, China; 2 Guangdong Provincial Key Laboratory of Gastroenterology, Department of Gastroenterology, Nanfang Hospital, Southern Medical University, Guangzhou, Guangdong, China; Saint Louis University, United States of America

## Abstract

The incidence of hypertriglyceridemia induced acute pancreatitis (HTAP) continues to rise in China. It has systemic complications and high mortality, making the early assessment of the severity of this disease even more important. Circulating microRNAs (miRNAs) could be novel, non-invasive biomarkers for disease progression judgment. This study aimed to identify the potential role of serum miRNAs as novel biomarkers of HTAP progression. HTAP patients were divided into two groups: moderately severe (HTMSAP) and severe (HTSAP), healthy people were used as control group. The serum miRNA expression profiles of these three groups were determined by microarray and verified by qRT-PCR. The functions and pathways of the targeted genes of deregulated miRNAs were predicted, using bioinformatics analysis; miRNA-mRNA network was generated. Moreover, the correlation between miR-181a-5p and pancreatitis metabolism related substances were studied and the serum concentration of inflammatory cytokines and miRNAs at different time points during the MSAP and SAP were investigated, respectively. Finally, the receiver operating characteristic (ROC) curve of miRNAs was studied. Significant changes in the serum concentration of the following miRNAs of HTAP patients (P<0.05) were discovered: miR24-3p, 361-5p, 1246, and 222-3p (constantly upregulated), and 181a-5p (constantly downregulated) (P<0.05). Bioinformatics analysis predicted that 13 GOs and 36 pathways regulated by overlap miRNAs were involved in glucose, fat, calcium (Ca++), and insulin metabolism (P<0.001). miRNA-mRNA network revealed that the overlap miRNAs targeted genes participating in pancreas metabolism and miR-181a-5p, the only downregulated miRNA, had good negative correlation with triglyceride (TG), total cholesterol (TC), and fast blood glucose (FBG), but a positive correlation with Ca++. When compared with inflammatory cytokines, the changes of all five overlap miRNAs were more stable. It was found that when used for evaluating the progression of HTAP, miRNAs showed good AUC. These data suggested that serum miRNAs have the potential to be excellent HTAP biomarkers.

## Introduction

Hypertriglyceridemia (HT) is the third most common cause of AP following gallstones and alcohol. It is a potentially life-threatening disease with a wide spectrum of severity, the overall mortality of AP is approximately 5% and the mortality of severe AP patients is up to 10% or even higher [1]. Although the association between HT and AP is well established and the risk and relative burden of AP in patients with differing degrees of HT were studied [2], there is still no ideal biomarker for early assessment of the severity of this disease presently.

Recent studies show that miRNAs in serum or plasma can be stably detected and used as diagnostic and prognostic markers in diseases [3]: circulating miRNAs were deregulated in pancreatic cancer, and thousands of miRNAs have been screened in pancreatic cancer, several of which could have diagnostic utility [4]. However, the changes of circulating miRNAs in HTAP are unclear. Gene ontology (GO) and the kyoto encyclopedia of genes and genomes (KEGG) are bioinformatics databases used for computational prediction of highly complex cellular processes and organism behavior, including signaling pathways and gene function [5]. In our previous study, bioinformatics have been successfully used to predict miRNA function, signaling pathways, and gene networks in acute liver failure [6].

It is still unclear whether the profile of serum miRNAs is deregulated during the development of HTAP. In the current study, we report a possible role of circulating miRNAs as biomarker in the progression of HTAP.

## Results

### Serum miRNAs expression profile was significantly changed in HT related MSAP and SAP patients

**Figure 1:**
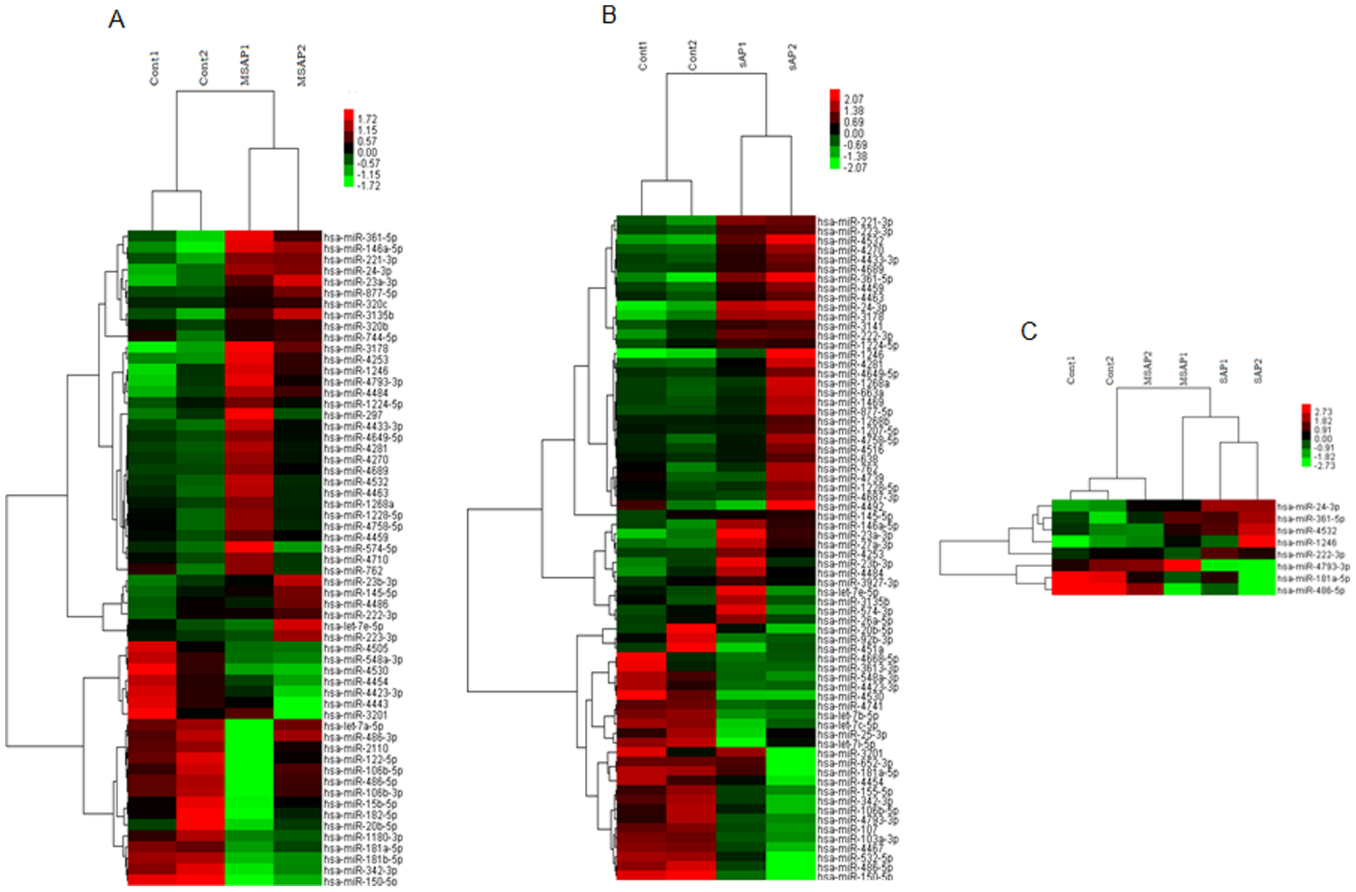
Microarray analysis of the serum miRNA expression profile of control MSAP and SAP patients. The expression profile of serum miRNAs was detected, using the LAN-based miRNA microarray. (A) There were 59 deregulated serum miRNAs (P<0.01) in MSAP patients and (B) 70 deregulated serum miRNAs (P<0.01) in SAP patients, compared with the healthy control group. (C) Among these deregulated miRNAs, eight were overlap miRNAs between the two AP groups. The top bar represents the signal levels of miRNA expression from −1.72 (green) to 2.73 (red). The individual identity of the significantly deregulated miRNAs is shown on the right border.

Microarray revealed that there were 59 deregulated serum miRNAs (P<0.01) in MSAP patients, compared with the healthy control group, of which 37 were upregulated and 22 were downregulated (P<0.01) (Figure 1A). In SAP patients, there were 70 deregulated serum miRNAs (P<0.01), compared with the healthy control group, of which 44 were upregulated and 26 were downregulated (P<0.01) (Figure 1B). Evidently, eight deregulated miRNAs were overlap between the two HTAP groups (P<0.05) (Figure 1C).

### The expression of miRNAs were verified by RT-PCR

**Figure 2:**
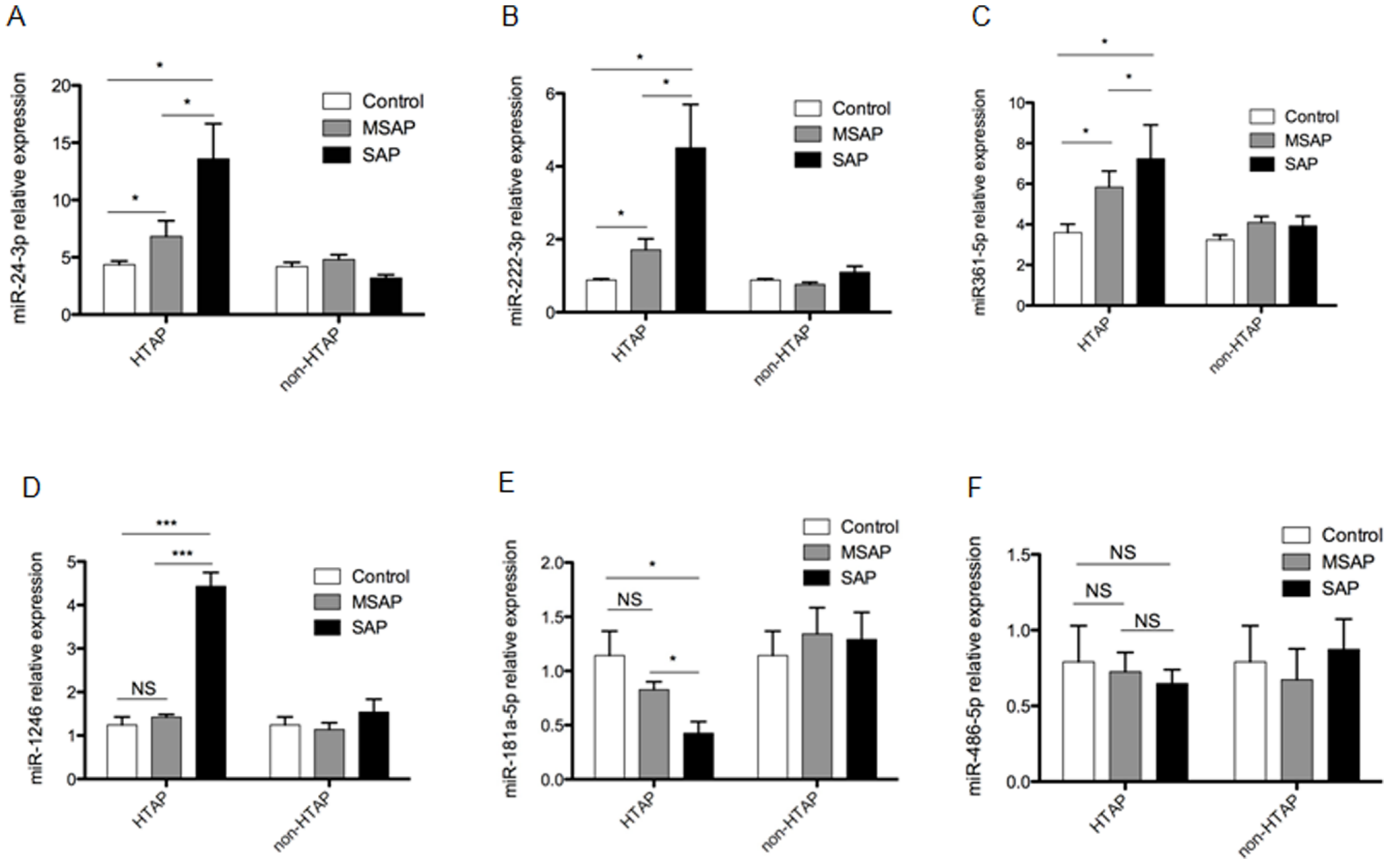
Verification the expression of overlap miRNAs via RT-PCR. The expression of eight overlap miRNAs was verified via qRT-PCR. The x-xis represents the different groups (HTAP: hypertriglyceridemia induced acute pancreatitis, non-HTAP: acute pancreatitis caused by factors other than hyperlipidemia), and the y-axis represents the relative expression of (A) miR-24-3p, (B) miR-222-3p, (C) miR-361-5p, (D) miR-1246, (E) miR-181a-5p, and (F) miR-486-5p respectively. Mean ±SEM. *P<0.05, **P<0.01, *** P<0.001, NS: no significant changes.

Five of the eight overlap miRNAs mentioned above were verified by RT-PCR. Four of them (miR24-3p, 222-3p, 361-5p, and 1246) were overexpressed in both the two HTAP groups (MSAP and SAP), compared with the healthy control group, (Figure 2A, B, C, D) (P<0.05). Only one, miR-181a-5p, was downregulated in two HTAP groups (Figure 2E) (P<0.05). The change of miR-486-5p was not significant in any of the three groups (Figure 2F) (P>0.05), and the expression of miR-4532 and miR-4793-3p were too low to be detectable in two HTAP groups (data not show). The expressions of these eight miRNAs were also examined in non-HTAP group, and it was found that compared with healthy control, none of the miRNAs exhibited significantly different expression in non-HTAP group (P>0.05).

### Microarray based bioinformatics prediction revealed the role of overlap miRNAs in HTAP

**Figure 3:**
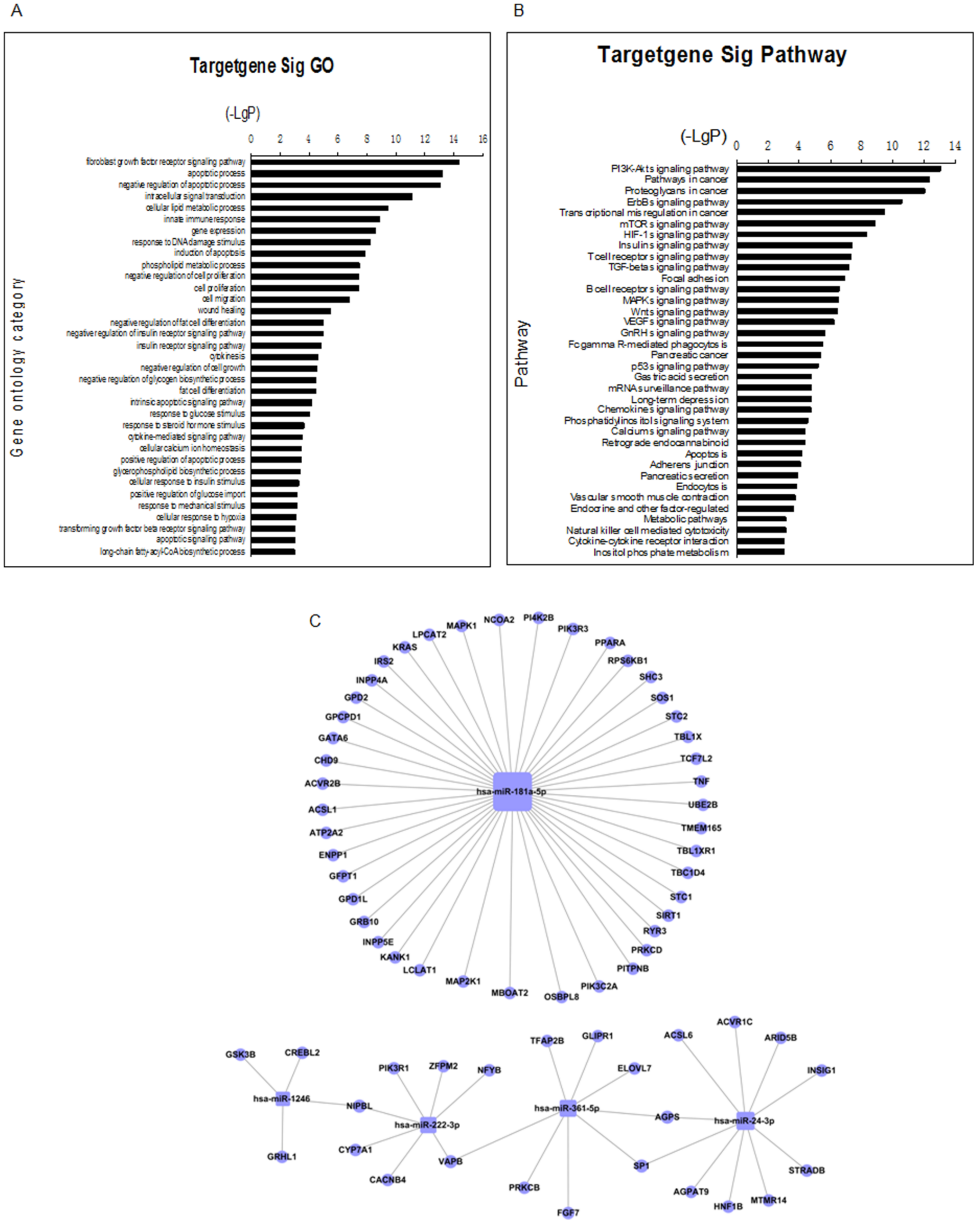
Prediction of functions and signal pathways of overlap miRNAs. (A) miRNA-GO network was generated according to the relationships between significant functions and 5 overlap miRNAs. The vertical axis is the GOs category and the horizontal axis is the P value of each GO. The lower the P value was, the more miRNAs regulated the GOs and the more important was the roles of GOs in HTAP. (B) miRNA-KEGG-network was generated according to the relationship between significant signal pathways and 5 overlap miRNAs. The vertical axis is the pathways category and the horizontal axis is the P value of each pathway. The lower the P value was, the more miRNAs regulated the pathways and more important was the roles of GOs in HTAP. (C) miRNA–mRNA-Network. The blue circles represent genes, and the blue squares represent miRNAs; lines represent the relationship between miRNA and genes. The network is used to assess the regulatory status of miRNAs and genes. The degree (size) of the blue square correlates with the regulatory functionality of miRNA; i.e., the bigger the degree, the more functions of the miRNAs.

It was demonstrated that 35 GOs were regulated significantly (P<0.01) by these five overlap miRNAs (Figure 3A). Among these GOs, 13 GOs were related to metabolic processes of glucose, fat, calcium, and insulin, including the following: cellular lipid metabolic process, negative regulation of fat cell differentiation, insulin receptor signaling pathway, fat cell differentiation, response to glucose stimulus, cellular response to insulin stimulus, positive regulation of glucose import, long-chain fatty-acyl-CoA biosynthetic process (Table S2).

The KEGG predicted that 36 pathways were regulated significantly (P<0.01) by these five overlap miRNAs (Figure 3B). Many of these signaling pathways such as pancreatic secretion, calcium reabsorption (regulated by either hormones or other factors), calcium signaling pathway, gastric acid secretion, and insulin signaling pathway have been shown to participate in the progression of AP (Table S3).

To illustrate the relationship between overlap miRNAs and their targeted genes, the miRNA-mRNA network was generated, based on the GO and the KEGG predicted data (see above). Target scan (http://www.targetscan.org/) revealed that the overlap miRNAs targeted genes that participated in the fat, glucose, insulin or calcium metabolism and which have been found to play key roles in HTAP [7] (Figure 3C, Table S4).

### miR-181a-5p had a close correlation to TG, TC, Ca++, FBG and inflammatory cytokines

**Figure 4:**
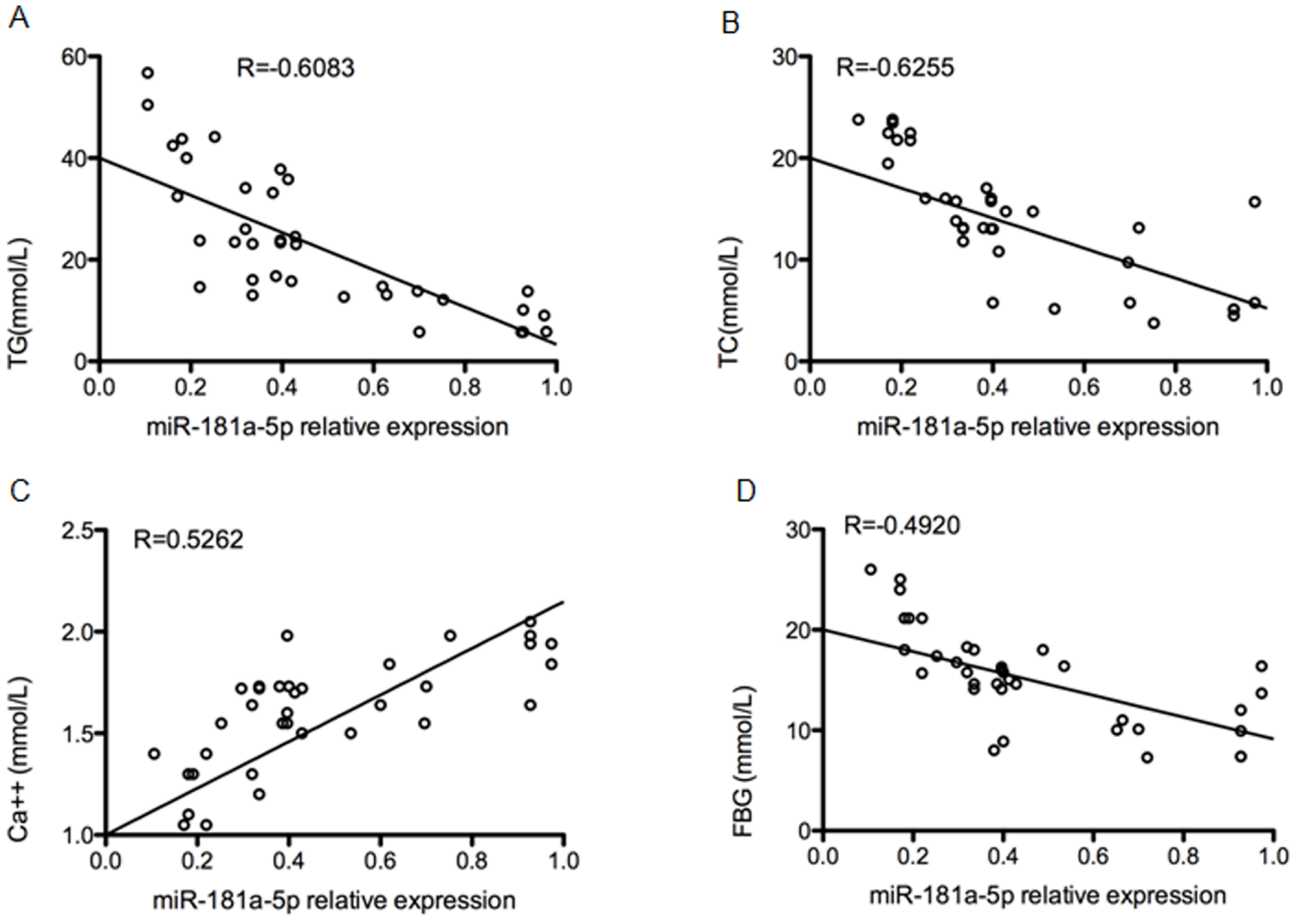
miR-181a-5p had a close correlation with TG, TC, Ca++ and FBG respectively in HTSAP. The correlation between the expression of miR-181a-5p and that of (A) triglycerides (TC), (B) total cholesterol (TC), (C) calcium (Ca++), and (D) fasting blood glucose (FBG) were examined. R represents a correlation coefficient; a negative R value suggests a negative correlation and a positive R value a positive correlation; Mean ±SEM.

The above-mentioned miRNA-mRNA bioinformatics analysis showed that the enrichment degree of the down-regulated miR-181a-5p was the largest, indicating its deeper involvement in the regulation of genes associated with the metabolism of fat, insulin, glucose, and calcium. Further correlation analysis revealed that miR-181a-5p was significantly and negatively correlated with TG, TC, and FBG, with the correlation coefficient R being −0.6083, −0.6255, and −0.4920, respectively; miR-181a-5p was significantly and positively correlated with Ca++ and the correlation coefficient R was 0.5262 (P<0.01) (Figure 4A, B, C, D).

In order to investigate the relationship between miRNAs and inflammatory cytokines in the HTAP, we studied the correlation between miR-181a-5p and inflammatory cytokines PCT, IL-1βand IL-6, it was disclosed that miR-181a-5p had significant and negative correlation with PCT, IL-1βand IL-6, with the correlation coefficient R being −0.6783, −0.6294, and −0.5849, respectively (Figure S2A,B,C).

### The dynamic expression of overlap miRNAs and inflammatory cytokines at different time points in HTAP

**Figure 5:**
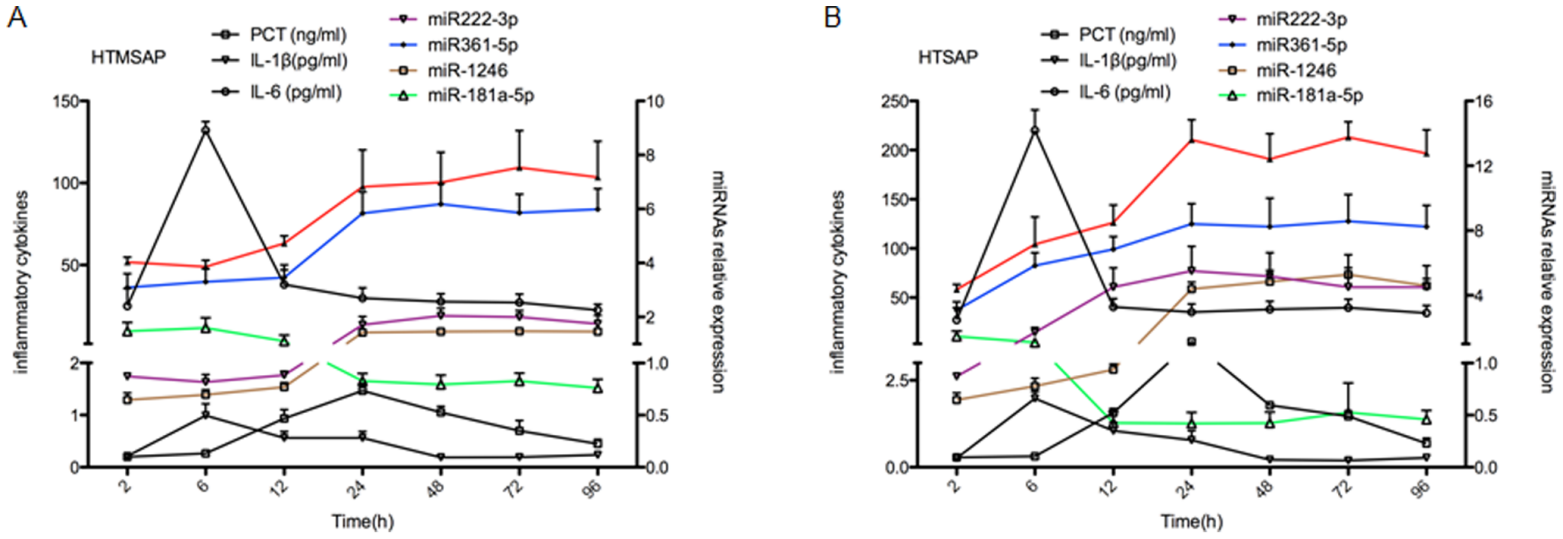
Expression of miRNAs and inflammatory cytokines at different time points during HTAP. The levels of serum inflammatory cytokines and the five overlap miRNAs at time points of 2, 5, 12, 24, 48, 72, and 96 hours after (A) HTMSAP and (B) HTSAP onset were examined. In this figure, miRNAs expressions were shown using curves of different colours. The abscissa represents the time interval between disease onset and sample collection; the left vertical axis represents the level of serum inflammatory cytokines, and the right vertical axis represents the relative expression of the five overlap miRNAs.

Furthermore, the dynamic expression of the overlap miRNAs and inflammatory cytokines was studied. The serum samples of the 22 cases of MSAP and 18 cases of SAP patients were collected at different time points after disease onset, and the levels of miRNAs as well as inflammatory cytokines (PCT, IL-1β and IL-6) were measured. The results showed that in the HTMSAP group, IL-6 expression rose rapidly, reached its peak at six hours after the disease onset, and then rapidly declined after another six hours. The dynamic change in IL-1β was similar to that of IL-6. PCT expression started to rise six hours after the disease onset, reached its peak value at 24 hours after the disease onset, but declined to a very low level at 72 hours after the disease onset. The expression of the five miRNAs changed significantly after 12 hours of the disease onset (increase or decrease), reached maximal values at 24 hours of the disease onset, and remained stable afterwards (Figure 5A). The dynamic expression of miRNAs and inflammatory cytokines was also studied in HTSAP group, they showed the similar changes (Figure 5B).

### Diagnostic Accuracy of Serum overlap miRNAs of HTAP Severity

**Figure 6:**
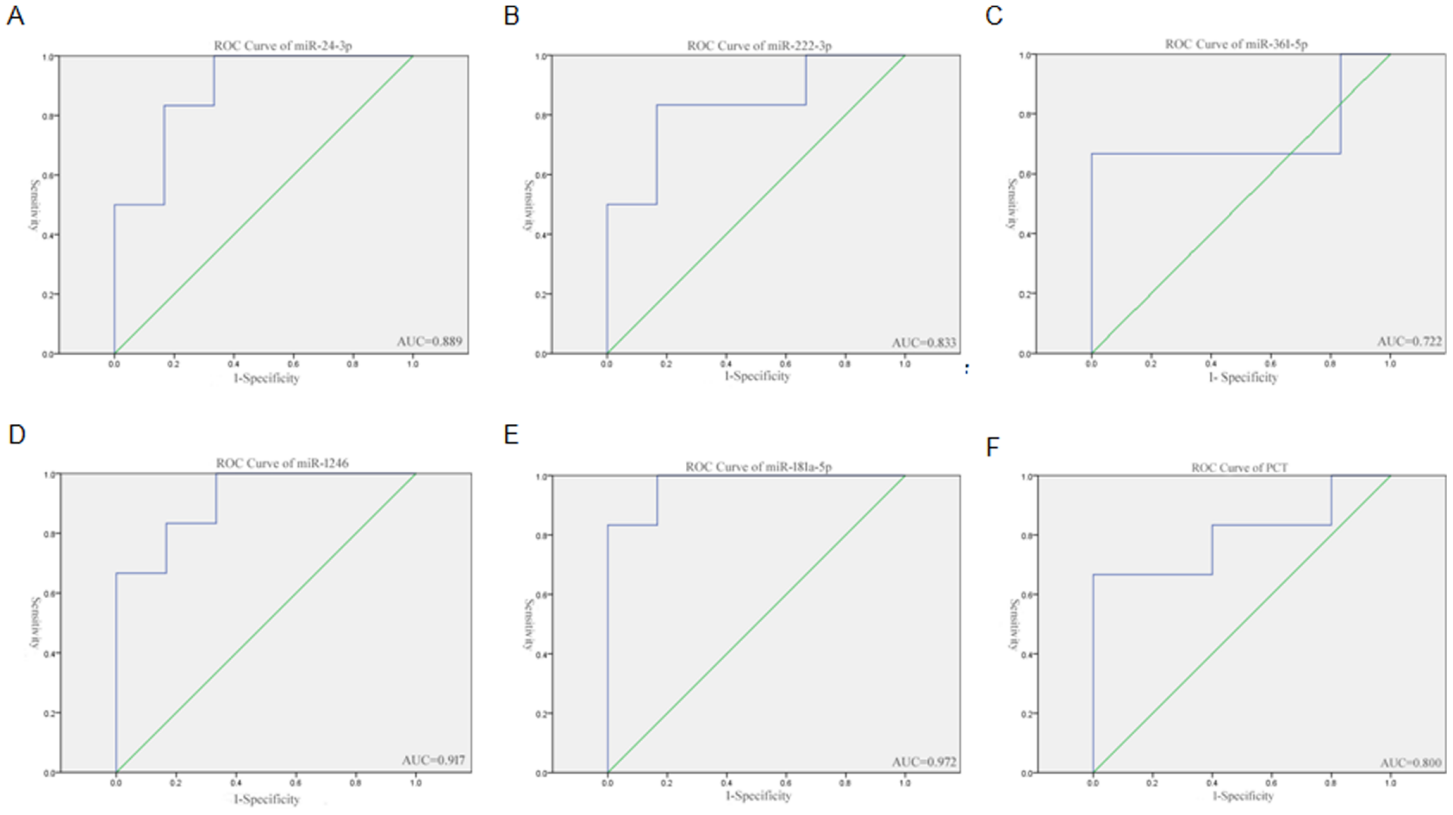
Receiver operating characteristic (ROC) curve analysis of overlap miRNAs for the judgment of HTAP severity. ROC analysis allows the evaluation of the binary classification variable (MSAP  = 0, SAP  = 1) at different thresholds of miRNA levels. The area under the curve (AUC) was calculated. The 95% confidence interval (CI) is therefore a measure for the reliability of miRNA-based HTAP severity diagnosis.

In order to investigate the sensitivity and specificity of these serum microRNAs as diagnostic markers of the HTAP severity, a receiver operating characteristic (ROC) curve analysis was performed. The results showed that the area under the curve (AUC) for miR24-3p was 0.889 (95% confidence interval (CI)  = 0.697–1.0; sensitivity: 100%; specificity: 83.3%; P<0.01) (Figure 6A). The AUC for miR-222-3p was 0.833 (95% CI = 0.590-1.0; sensitivity: 100%; specificity: 83.3%; P<0.01) (Figure 6B). The AUC for miR-361-5p was 0.722 (95% CI = 0.96–1.0; sensitivity: 85%; specificity: 90%; P<0.01) (Figure 6C). The AUC for miR-1246 was 0.917 (95% CI = 0.673–0.95; sensitivity: 98%; specificity: 92%; P<0.01) (Figure 6D). The AUC for miR-181a-5p was 0.972 (95% CI: 0.604–1.0; sensitivity: 100%; specificity: 83.3%; P<0.01) (Figure 6E). Meanwhile, the ROC curve analysis for PCT was performed, the AUC for PCT was 0.800 (95% CI = 0.520–1.0; sensitivity: 100%; specificity: 80%; P<0.01) (Figure 6F). Thus it can be seen under the similar sensitivity and specificity, PCT with an AUC value of 0.800, which was not found to be superior to serum miRNAs. It was speculated that the expression of the above five miRNAs, especially miR-181a-5p, can be used to accurately evaluate the progression of HTAP.

## Discussion

According to the current state of knowledge, miRNAs circulate in the bloodstream and other bodily fluids in a very stable, cell-free form and may serve as a novel diagnostic marker [8]. In this study, we detected the serum miRNAs of HTAP patients with different disease severity. Our data showed that serum miRNAs were significantly changed in both MSAP and SAP patients. Five of these deregulated miRNAs are overlap from MSAP to SAP groups (consistently upregulated or downregulated). However, this phenomenon was not found in the non-HTAP patients. Our novel findings suggest that the five mRNAs may be one of the factors determining the severity of the HTAP. In this study, we also checked the five overlap miRNAs expression in HT induced mild acute pancreatitis (HTMAP) patients by RT-PCR, and discovered (data not shown) that they have no significant changes, compared with the healthy control (data not show). Therefore we focused our studies on the differences between MSAP and SAP patients in HTAP.

The GO annotations have proven to be remarkably useful to determine the significance of microarray results concerning the functional significance [9]. In this study, based on the microarray data, GO analysis was applied to predict the roles of these five overlap miRNAs in the progression of HTAP. It was discovered that many GOs regulated by the miRNAs were related to glucose, fat calcium and insulin metabolic processes, including negative regulation of fat cell differentiation, insulin receptor signaling pathway, fat cell differentiation, response to glucose stimulus, cellular response to insulin stimulus, and positive regulation of glucose import and long-chain fatty-acyl-CoA biosynthetic process.

The KEGG is widely used for analysis of various types of molecular biological data in order to discover higher-order functions [10], [11]. We examined the roles of miRNAs regulated pathways involved in the development of HTAP in processes such as pancreatic secretion, calcium reabsorption (regulated by either hormones or other factors), calcium signaling pathway, gastric acid secretion, and insulin signaling pathway.

In order to further investigate the roles of deregulated miRNAs in the development of HTAP, miRNA-mRNA interactions were explored; for this purpose, miRNA-mRNA networks were employed as they efficiently detect mRNAs deregulated by miRNAs [12]. It was predicted that five overlap miRNAs targeted genes involved in fat/lipid metabolic process, insulin metabolism, glucose/glycogen metabolism, and cellular calcium ion homeostasis.

Pancreatic lipase hydrolyses TG to generate free fatty acids (FFA) [13], The degradation of lipoprotein to FFA may produce a proinflammatory response, further damaging the acinar structure of pancreatic cells [14]. It has been reported that FFA is involved in the development of complications in acute pancreatitis [15]. FFA serum levels in acute necrotizing pancreatitis were significantly higher than in acute edematous pancreatitis, and serum FFA correlated positively with C-reactive protein levels. In acute necrotizing pancreatitis, the higher and sustained elevation of FFA may predominantly response the pancreatic parenchymal and extrapancreatic fat necrosis [16]. In the present study, it was found that the TC and TG levels of the two HTAP groups were both significantly increased, and their levels in the HTSAP group (approximately 13.38 mmol/l and 24.47 mmol/l, respectively) were higher than that in the HTMSAP group (approximately 2.9 mmol/l and 12.85 mmol/l, respectively). This suggests that in the HTSAP patients more FFA is produced than in the HTMSAP patients, leading to necrosis of the pancreas and the surrounding adipose tissues.

Many studies found that Ca++ is overloaded in pancreatic acinar cells [17], the sustained calcium elevation activates intracellular digestive proenzymes which leads to necrosis and toxicity [18]–[20], resulting in necrosis and inflammation in AP [21], [22]. Based on these previous findings, in the present study we did not focus on examining the calcium content in the pancreatic acinar cells in the pancreatic tissue of AP using animal experiments, but rather focused on investigating the relationship between the miRNAs and the calcium expression. It was found that in AP the serum Ca++ is decreased because of “saponification” and “hypoproteinemia” theories, etc. In the current study, we found the serum concentration of Ca++ in HTSAP group (about 1.48 mmol/l) was much lower than in HTMSAP groups (about 1.89 mmol/l). The key pancreatic digestive enzymes include trypsin, lipase and amylase, in this study, it was found the level of these three digestive enzymes were significantly elevated in both HTMSAP and HTSAP groups, and the level of Try-II in HTSAP group was much higher than in HTMSAP groups (P<0.05). In conclusion, it was speculated that there is more auto-digestion and necrosis in HTSAP cases than in HTMSAP cases [23]. We measured the FBG concentration of HTAP patients, and could demonstrate that its upregulation was more significant in SAP patients compared with MSAP patients.

Bioinformatics analysis revealed that among these miRNAs, downregulated miR-181a-5p showed the highest enrichment degree. It was speculated that miR-181a-5p plays key roles in the progression of HTAP by targeting the genes involved in pancreatic metabolism. Further correlation analysis showed that miR-181a-5p exhibited a significant correlation with TG, TC, Ca++, and FBG. At present, we are exploring further regulatory mechanisms of miR-181a-5p concerning HTAP.

Inflammatory cytokines such as interleukin-1β (IL-1β) and interleukin-6 (IL-6) play key roles in acute pancreatitis at the early stage of HTAP [24], [25]. Procalcitonin (PCT) is the protein that is valuable in the prognosis of AP [26]. In the present experiment, it was found that, miR-181a-5p had good negative correlation with PCT, IL-1βand IL-6. Many studies showed cytokines can regulate the miRNAs transcription through different signaling pathways [27], it is possible that the inflammatory cytokines can regulate the overlap miRNAs expression during HTAP. At present, we are doing further experiment to obtain the possibility of the regulatory role of inflammatory cytokines to miRNAs expression.

Although the IL-1β and IL-6 expressions began to increase early after HTAP (within six hours), after another six hours, the IL-1β and IL-6 expression levels declined sharply and were close to normal at 24 hours after disease onset. They showed short half-life [28]. PCT reached the highest value 24 hours after the disease onset, but afterwards its level also declined dramatically. The expressions of the five overlap miRNAs changed significantly after 12 hours of the disease onset (up or down regulation), then they reached a plateau at time point of 24 hours and remained stable until the last time point of examination (96 hours). The severity of HTAP patients turned from MSAP to SAP generally after 24 hours of disease admission. Therefore, it was speculated that the expression levels of these inflammatory factors can not reflect the progression and outcome of the HTAP satisfactorily because of their short half-life.

Usually, the early prediction of the AP can be made by scoring systems such as “Ranson”, but it needs 48 hours of disease onset to complete the assessment and is uncomfortable to use everyday. At present, a great attention is still given for seeking a single prognostic biomarker. The most widely explored and described single predictor is PCT [29], but some studies found PCT is of limited additional value for early assessment of severity in AP [30]. Molecular biomarkers of a disease do not only have to be tissue-specific, but also highly stable and detectable. Nowadays, almost all of the routinely used serum markers are proteins and the conventional methodologies used to measure them remain labor-intensive. Moreover, miRNAs are present in human plasma in a remarkably stable form, and are resistant to RNaseA digestion and other harsh conditions. The serum miRNAs levels are stable, reproducible, and consistent among individuals of the same animal species [31]–[33]. They are stable in almost every bodily fluid and their presence and level is related to the severity and progression of diseases [34], although the mechanisms are not clear [8], [35], [36]. Based on the studies above, further the analysis on the sensitivity and specificity of the miRNAs as molecular biomarkers revealed that the above five overlap miRNAs, especially miR-181a-5p, with the similar sensitivity and specificity, exhibited relatively high AUC in evaluating the progression of HTAP from MSAP to SAP when compared with PCT. However, it needs further studies to get a perfect reference value as a diagnostic cutoff point.

Tissue-specific serum miRNA profiling would be of interest in the future. However, we did not find any literature on the tissue source of these five miRNAs in the PubMed database. Currently, effective methods for determining the tissue source of miRNAs are still lacking, and the tissue source of miRNAs can only be inferred from the relative expression levels of miRNAs in the relevant organs and tissues. Therefore we are currently in the process of establishing an HTAP animal model, in order to obtain the tissue source of the five overlap miRNAs, particularly miR-181a-5p. The results will be reported in future studies.

In conclusion, our results suggest that miRNAs especially miR-181a-5p play important roles in the progression of HTAP. These findings may provide useful data in the development of a therapeutic strategy and find new HTAP biomarkers.

## Methods

### Ethics Statement

The current study was approved by the Nanjing Medical University Clinic Institutional Review Board (IRB) protocols. All patients provided written informed consent prior to study commencement.

### The diagnosis of HTAP and the severity criteria of HTAP

The diagnosis of HT is made if the TG level is greater than 11.3 mmol/l [37], or the TG level is between 5.65 and11.3 mmol/l and the serum is milky. At least two of the following three conditions must be met for the diagnosis “AP” to be valid: (1) persistent upper abdominal pain; (2) serum lipase or amylase increase to at least 3 times of normal level; and (3) characteristics of AP in abdominal CT. The diagnosis of HTAP is caused by hyperlipidemia only and based on the clinical manifestations of both HT and AP.

The severity of HTAP was defined according the 2012 Mayo clinic criteria for assessing severity of AP [38]: The classification defines three degrees of severity (MAP, MSAP, and SAP). MAP: no organ failure and lack of local or systemic complications. MSAP: organ failure that resolves within forty-eight hours (transient organ failure) and/or local or systemic complications (sterile or infected) without persistent organ failure. SAP: persistent single or multiple organ failures (longer than forty eight hours).

By comparison, non-HTAP is caused by factors other than hyperlipidemia, such as gallstones and alcohol. The clinical characteristics and abdominal CT manifestations of the HTAP patients are shown in Table S1 and Figure S1A, B, C, D.

### Human serum collection

We collected the blood samples of 82 healthy controls at the healthy physical examination center of Wuxi People's Hospital. Total HTAP serum samples were obtained from 79 patients at different time points after admission to the emergency room at the Wuxi People's Hospital. Among these samples, 43 were from MSAP and 36 from SAP patients. We also collected serum samples from 118 AP patients without hypertriglyceridemia (non-HTAP) at different time points after admission to the emergency room at the Wuxi People's Hospital. Among these samples, 63 were from MSAP and 55 from SAP patients. All blood specimens were maintained in collection tubes with no additives for 2 hours at 20 to 22°C and then centrifuged (Minifuge RF, Heraeus, Hanover, Germany) at 1200×g and 4°C for 20 mins. Serum was separated and snap frozen upon acquisition and stored at −80°C until use.

### Serum RNA extraction and miRNA microarray

The circulating miRNAs study followed the guidelines of relevant literature [39]. The serum specimens were collected 24 hours after HTAP onset. Total RNA was extracted from frozen serum, using QIAGEN RNeasy Mini Kit (217004; QIAGEN, Germany) according to the manufacturer's instructions, as we described in our previous work [6]. Small isolated RNAs were extended and hybridized with fluorescence labeled with biotin dyes on a Gene Chip miRNA 3.0 Array (Affymetrix, Cleveland, OH, USA). Following hybridization, the images were digitized and analysed using a laser scanner interfaced with ArrayPro image analysis software (Media Cybernetics, Silver Spring, MD, USA). The microarray data have been submitted to ArrayExpress and the accession number is E-MTAB-2642.

### Quantitative real-time RT-PCR (qRT-PCR)

The expression of mRNAs was confirmed, using qRT-PCR according to the manufacturer's instructions as we described in our previous work [6]. cDNA was synthesized from total RNA obtained above, using a Quantscript reverse transcription kit (Tiangen Biotech Beijing CO., LTD., China). qRT-PCR was performed with the SYBR Green SuperReal PreMix (Tiangen Biotech Beijing CO., LTD., China) with an ABI 7,500 qPCR system (Applied Biosystems, Foster City, CA, USA). Optimal dilution and melting curves were utilised for specificity of amplified production for each primer set. Each sample was diluted serially over three orders of magnitude. qPCR was performed to detect human miR24-3p, 222-3p, 361-5p, 1246, 181a-5p, and 486-5p, while U6 was utilized as an internal control for miRNAs, The relative amount of each gene was measured using the 2(-DDCT) method [40]. All qRT-PCR reactions were performed in triplicate and repeated three times.

### Bioinformatics analysis

GO analysis was applied to analyze the main function of the differential expression genes which can organize genes into hierarchical categories and uncover the gene regulatory network on the basis of biological process and molecular function [5]. Pathway analysis was used to find out the significant pathway of the differential genes according to KEGG, Biocarta and Reatome [10], [41]. The relationship between miRNAs and genes was counted by their differential expression values and according to their interactions in the Sanger miRNA database. [42] We described in detail the methods of bioinformatics analysis in our previous work [6].

### Clinic biochemistry parameters of HTAP patients

The serum samples of MSAP and SAP patients were collected 24 hours after HTAP onset, the levels of alanine transaminase (ALT), aspartate transaminase (AST), albumin (ALB), TG, FBG, Ca++, serum amylase (AMY) and lipase (LPS) were determined using a enzymatic method on a standard biochemical autoanalyzer (Beckman coulter UniCel DxC 800, Fullerton, CA, USA) according to the manufacturer's instructions. The level of Try-II was detected via enzyme linked immunosorbent assay (ELISA) according to the manufacturer's instructions (B&D Biosciences; San Diego, CA, USA), all experimental samples were tested in duplicate.

### The concentration of serum inflammatory cytokines were analyzed

The serum of control, MSAP and SAP groups were collected 6 hours after HTAP onset, the concentrations of IL-1β and IL-6 were determined via ELISA according to the manufacturer's instructions (B&D Biosciences; San Diego, CA, USA), all experimental samples were tested in duplicate. The radioimmunoassay was carried out to detecte the serum levels of the PCT 24 hours after the disease admission according to the manufacturer's instructions (B&D Biosciences; San Diego, CA, USA). The assay is based upon the competition of labeled ^125^I-peptide and unlabeled peptide binding to the limited quantity of antibodies specific for the standard peptide in each reaction mixture.

### The correlation between miR-181a-5p and pancreatic metabolism-related factors as well as inflammatory cytokines were analyzed

The relationship between miR-181a-5p and factors related to pancreatic metabolism such as TG, TC, Ca++ and HBG as well as inflammatory cytokine PCT were studied 24 hours after HTSAP onset using spearman's correlation coefficient. Meanwhile, the relationship between miR-181a-5p and IL-1βand IL-6 were studied 6 hours after HTSAP onset using spearman's correlation coefficient.

### Statistical analysis

All data are presented as means ±SEM. One-way or two-way ANOVA was performed for statistical analysis, using GraphPad Prism 5. Differences between group means with P values <0.05 were regarded as being statistically significant. The correlations were analyzed by Pearson correlation coefficients. Receiver operating characteristic (ROC) curve analysis was performed using SPSS19.0.

## Supporting Information

Figure S1 **CT manifestations of MSAP and SAP patients.** A 35-year-old woman with HTMSAP. (A) The CT scan showed a pancreatic volume increase, necrosis of the tail of the pancreas, and large amounts of fluid density shadows (white arrow) surrounding the pancreas one week of disease onset. (B) A week later, the CT scan revealed that pancreatic necrosis was, compared with before, notably absorbed and was narrowing (white arrow). A 28-year-old woman with HTSAP. (C) The CT scan showed a pancreatic volume increase and flocculent water-like density shadows in the surrounding area one week of disease onset. (D) A week later, the CT scan revealed an increased pancreatic volume, flocculent water-like density shadows in the surrounding area (white arrow), and there was also pancreatic pseudocyst formation (white stars). These results suggest that patients with SAP have more severe outcome.
(TIF)

Figure S2 **The correlation between the miR-181a-5p and inflammatory cytokines in HTSAP.** The correlations between miR-181a-5p and inflammatory cytokines (A) PCT, (B) IL-1β and (C) IL-6 were studies, X axis represents miR-181a-5p, and Y axis represents the levels of inflammatory cytokines. The correlations were analysed by Pearson correlation. “R” represents correlation coefficient. “-” represents negative correlation.
(TIF)

Table S1 **Clinical characters of HTAP.** The table shows the relevant clinical data of HTAP patients. It can be seen that, compared with the MSAP group, in the SAP group the levels of the biochemical markers TG, TC, Ca, FG, ALT, and AST, as well as the levels of the inflammation-related factors IL-6, IL-1β, and PCT changed significant (P<0.05), whereas age and ALB did not differ significantly between the two groups. The amylase, lipase and trypsinogen-II were elevated obviously in both MSAP and SAP groups, and the level of Try-II in SAP group was much higher than in MSAP groups (P<0.05). Among total 36 SAP patients, there are 20 patients with heart injury, 25 patients with lung injury and 8 patients with kidney injury over 48 hours, conversely no patients have organ failure over 48 hours in MSAP group. The mortality rate of SAP is 11%, which is higher than MSAP (0). All data were presented with mean ±SEM; differences between MSAP and SAP means with P values greater than 0.05 were regarded as statistically significant.
(DOC)

Table S2 **GOs and involved genes targeted by overlap miRNAs.** The false discovery rate (FDR) was calculated to correct the P value. Enrichment degree means the contribution of miRNAs to the GOs. The key functions in the network always have the higher enrichment degrees (P<0.01).
(DOC)

Table S3 **Pathways and genes targeted by overlap miRNAs.** The false discovery rate (FDR) was calculated to correct the P value. Enrichment degree means the contribution of miRNAs to the signal pathways. Key signal pathways of the network always have higher enrichment degrees (P<0.01).
(DOC)

Table S4 **Overlap miRNAs targeting gens involved in fat, glucose, insulin, calcium metabolize.** Based on the data above, the targeted genes related to pancreas metabolism were showed.
(DOC)

## References

[1] Karpavicius A, Dambrauskas Z, Sileikis A, Vitkus D, Strupas K (2012) Value of adipokines in predicting the severity of acute pancreatitis: comprehensive review. World J Gastroenterol 18: 6620–6627. 23236237 10.3748/wjg.v18.i45.6620 PMC3516219

[2] Lloret Linares C, Pelletier AL, Czernichow S, Vergnaud AC, Bonnefont-Rousselot D, et al (2008) Acute pancreatitis in a cohort of 129 patients referred for severe hypertriglyceridemia. Pancreas 37: 13–12. 18580438 10.1097/MPA.0b013e31816074a1

[3] Berger F, Reiser MF (2013) Micro-RNAs as Potential New Molecular Biomarkers in Oncology: Have They Reached Relevance for the Clinical Imaging Sciences? Theranostics 3: 943–952. 24396505 10.7150/thno.7445 PMC3881096

[4] Du Y, Liu M, Gao J, Li Z (2013) Aberrant microRNAs expression patterns in pancreatic cancer and their clinical translation. Cancer Biother Radiopharm 28: 361–369. 23621126 10.1089/cbr.2012.1389

[5] Ashburner M, Ball CA, Blake JA, Botstein D, Butler H, et al (2000) Gene ontology: tool for the unification of biology. The Gene Ontology Consortium. Nat Genet 25: 25–29. 10802651 10.1038/75556 PMC3037419

[6] An F, Gong B, Wang H, Yu D, Zhao G, et al (2012) miR-15b and miR-16 regulate TNF mediated hepatocyte apoptosis via BCL2 in acute liver failure. Apoptosis 17: 702–716. 22374434 10.1007/s10495-012-0704-7

[7] Tsuang W, Navaneethan U, Ruiz L, Palascak JB, Gelrud A (2009) Hypertriglyceridemic pancreatitis: presentation and management. Am J Gastroenterol 104: 984–991. 19293788 10.1038/ajg.2009.27

[8] Kosaka N, Iguchi H, Ochiya T (2010) Circulating microRNA in body fluid: a new potential biomarker for cancer diagnosis and prognosis. Cancer Sci 101: 2087–2092. 20624164 10.1111/j.1349-7006.2010.01650.x PMC11159200

[9] Gene Ontology C (2006) The Gene Ontology (GO) project in 2006. Nucleic Acids Res 34: D322–326. 16381878 10.1093/nar/gkj021 PMC1347384

[10] Kanehisa M, Goto S, Kawashima S, Okuno Y, Hattori M (2004) The KEGG resource for deciphering the genome. Nucleic Acids Res 32: D277–280. 14681412 10.1093/nar/gkh063 PMC308797

[11] Yi M, Horton JD, Cohen JC, Hobbs HH, Stephens RM (2006) WholePathwayScope: a comprehensive pathway-based analysis tool for high-throughput data. BMC Bioinformatics 7: 30. 16423281 10.1186/1471-2105-7-30 PMC1388242

[12] Devaraj S, Natarajan J (2011) miRNA-mRNA network detects hub mRNAs and cancer specific miRNAs in lung cancer. In Silico Biol 11: 281–295. 23202429 10.3233/ISB-2012-0444

[13] Yang F, Wang Y, Sternfeld L, Rodriguez JA, Ross C, et al (2009) The role of free fatty acids, pancreatic lipase and Ca+ signalling in injury of isolated acinar cells and pancreatitis model in lipoprotein lipase-deficient mice. Acta Physiol (Oxf) 195: 13–28. 18983441 10.1111/j.1748-1716.2008.01933.x

[14] Nagayama D, Shirai K (2013) [Hypertriglyceridemia-induced pancreatitis]. Nihon Rinsho 71: 1602–1605. 24205721

[15] Sztefko K, Panek J (2001) Serum free fatty acid concentration in patients with acute pancreatitis. Pancreatology 1: 230–236. 12120200 10.1159/000055816

[16] Domschke S, Malfertheiner P, Uhl W, Buchler M, Domschke W (1993) Free fatty acids in serum of patients with acute necrotizing or edematous pancreatitis. Int J Pancreatol 13: 105–110. 8501351 10.1007/BF02786078

[17] Criddle DN, Gerasimenko JV, Baumgartner HK, Jaffar M, Voronina S, et al (2007) Calcium signalling and pancreatic cell death: apoptosis or necrosis? Cell Death Differ 14: 1285–1294. 17431416 10.1038/sj.cdd.4402150

[18] Wang Y, Sternfeld L, Yang F, Rodriguez JA, Ross C, et al (2009) Enhanced susceptibility to pancreatitis in severe hypertriglyceridaemic lipoprotein lipase-deficient mice and agonist-like function of pancreatic lipase in pancreatic cells. Gut 58: 422–430. 18936103 10.1136/gut.2007.146258

[19] Frick TW (2012) The role of calcium in acute pancreatitis. Surgery 152: S157–163. 22906890 10.1016/j.surg.2012.05.013

[20] Gerasimenko JV, Gerasimenko OV, Petersen OH (2013) The role of Ca2+ in the pathophysiology of pancreatitis. J Physiol. 10.1113/jphysiol.2013.261784 PMC3922492 23897234

[21] Orabi AI, Muili KA, Javed TA, Jin S, Jayaraman T, et al (2013) Cluster of differentiation 38 (CD38) mediates bile acid-induced acinar cell injury and pancreatitis through cyclic ADP-ribose and intracellular calcium release. J Biol Chem 288: 27128–27137. 23940051 10.1074/jbc.M113.494534 PMC3779711

[22] Huang G, Yao J, Zeng W, Mizuno Y, Kamm KE, et al (2006) ER stress disrupts Ca2+-signaling complexes and Ca2+ regulation in secretory and muscle cells from PERK-knockout mice. J Cell Sci 119: 153–161. 16352659 10.1242/jcs.02731

[23] Gerasimenko JV, Gryshchenko O, Ferdek PE, Stapleton E, Hebert TO, et al (2013) Ca2+ release-activated Ca2+ channel blockade as a potential tool in antipancreatitis therapy. Proc Natl Acad Sci U S A 110: 13186–13191. 23878235 10.1073/pnas.1300910110 PMC3740877

[24] de Beaux AC, Ross JA, Maingay JP, Fearon KC, Carter DC (1996) Proinflammatory cytokine release by peripheral blood mononuclear cells from patients with acute pancreatitis. Br J Surg 83: 1071–1075. 8869305 10.1002/bjs.1800830811

[25] Kusnierz-Cabala B, Gurda-Duda A, Dumnicka P, Kuzniewski M, Kulig J, et al (2013) [Analysis of selected inflammatory markers for early prediction of severe clinical course of acute pancreatitis]. Przegl Lek 70: 392–396. 24052976

[26] Gurda-Duda A, Kusnierz-Cabala B, Nowak W, Naskalski JW, Kulig J (2008) Assessment of the prognostic value of certain acute-phase proteins and procalcitonin in the prognosis of acute pancreatitis. Pancreas 37: 449–453. 18953261 10.1097/MPA.0b013e3181706d67

[27] Shi C, Zhu L, Chen X, Gu N, Chen L, et al (2014) IL-6 and TNF-alpha induced obesity-related inflammatory response through transcriptional regulation of miR-146b. J Interferon Cytokine Res 34: 342–348. 24428800 10.1089/jir.2013.0078 PMC4015473

[28] Volante E, Moretti S, Pisani F, Bevilacqua G (2004) Early diagnosis of bacterial infection in the neonate. J Matern Fetal Neonatal Med 16 Suppl 2: 13–16. 15590427 10.1080/14767050410001727116

[29] Rau BM, Kemppainen EA, Gumbs AA, Buchler MW, Wegscheider K, et al (2007) Early assessment of pancreatic infections and overall prognosis in severe acute pancreatitis by procalcitonin (PCT): a prospective international multicenter study. Ann Surg 245: 745–754. 17457167 10.1097/01.sla.0000252443.22360.46 PMC1877072

[30] Modrau IS, Floyd AK, Thorlacius-Ussing O (2005) The clinical value of procalcitonin in early assessment of acute pancreatitis. Am J Gastroenterol 100: 1593–1597. 15984987 10.1111/j.1572-0241.2005.41456.x

[31] Chen X, Ba Y, Ma L, Cai X, Yin Y, et al (2008) Characterization of microRNAs in serum: a novel class of biomarkers for diagnosis of cancer and other diseases. Cell Res 18: 997–1006. 18766170 10.1038/cr.2008.282

[32] Macha MA, Seshacharyulu P, Krishn SR, Pai P, Rachagani S, et al.. (2014) MicroRNAs (miRNA) as Biomarker(s) for Prognosis and Diagnosis of Gastrointestinal (GI) Cancers. Curr Pharm Des. 10.2174/1381612820666140128213117 PMC4113605 24479799

[33] Mitchell PS, Parkin RK, Kroh EM, Fritz BR, Wyman SK, et al (2008) Circulating microRNAs as stable blood-based markers for cancer detection. Proc Natl Acad Sci U S A 105: 10513–10518. 18663219 10.1073/pnas.0804549105 PMC2492472

[34] De Guire V, Robitaille R, Tetreault N, Guerin R, Menard C, et al (2013) Circulating miRNAs as sensitive and specific biomarkers for the diagnosis and monitoring of human diseases: promises and challenges. Clin Biochem 46: 846–860. 23562576 10.1016/j.clinbiochem.2013.03.015

[35] Hunter MP, Ismail N, Zhang X, Aguda BD, Lee EJ, et al (2008) Detection of microRNA expression in human peripheral blood microvesicles. PLoS One 3: e3694. 19002258 10.1371/journal.pone.0003694 PMC2577891

[36] Taylor DD, Gercel-Taylor C (2008) MicroRNA signatures of tumor-derived exosomes as diagnostic biomarkers of ovarian cancer. Gynecol Oncol 110: 13–21. 18589210 10.1016/j.ygyno.2008.04.033

[37] Cugnet-Anceau C, Moret M, Moulin P (2011) [Hypertriglyceridemia: therapeutic strategy]. Rev Prat 61: 1110–1116. 22135979

[38] Sarr MG, Banks PA, Bollen TL, Dervenis C, Gooszen HG, et al (2013) The new revised classification of acute pancreatitis 2012. Surg Clin North Am 93: 549–562. 23632143 10.1016/j.suc.2013.02.012

[39] Farina NH, Wood ME, Perrapato SD, Francklyn CS, Stein GS, et al.. (2013) Standardizing Analysis of Circulating MicroRNA: Clinical and Biological Relevance. J Cell Biochem. 10.1002/jcb.24745 PMC3992702 24357537

[40] Livak KJ, Schmittgen TD (2001) Analysis of relative gene expression data using real-time quantitative PCR and the 2(-Delta Delta C(T)) Method. Methods 25: 402–408. 11846609 10.1006/meth.2001.1262

[41] Draghici S, Khatri P, Tarca AL, Amin K, Done A, et al (2007) A systems biology approach for pathway level analysis. Genome Res 17: 1537–1545. 17785539 10.1101/gr.6202607 PMC1987343

[42] Joung JG, Hwang KB, Nam JW, Kim SJ, Zhang BT (2007) Discovery of microRNA-mRNA modules via population-based probabilistic learning. Bioinformatics 23: 1141–1147. 17350973 10.1093/bioinformatics/btm045

