# Supplementary figures and images for: From Moderately Severe to Severe Hypertriglyceridemia Induced Acute Pancreatitis: Circulating MiRNAs Play Role as Potential Biomarkers
Source: PLoS One. 2014 Nov 3;9(11):e111058. doi: 10.1371/journal.pone.0111058 (PMC4218837; doi:10.1371/journal.pone.0111058)

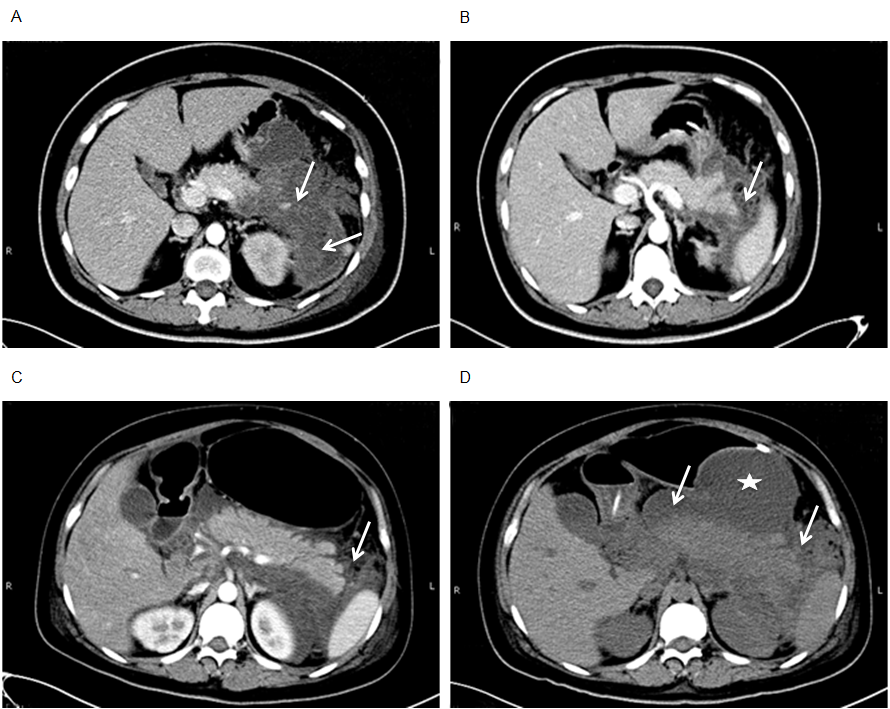

Supplement: Figure S1 — CT manifestations of MSAP and SAP patients. A 35-year-old woman with HTMSAP. (A) The CT scan showed a pancreatic volume increase, necrosis of the tail of the pancreas, and large amounts of fluid density shadows (white arrow) surrounding the pancreas one week of disease onset. (B) A week later, the CT scan revealed that pancreatic necrosis was, compared with before, notably absorbed and was narrowing (white arrow). A 28-year-old woman with HTSAP. (C) The CT scan showed a pancreatic volume increase and flocculent water-like density shadows in the surrounding area one week of disease onset. (D) A week later, the CT scan revealed an increased pancreatic volume, flocculent water-like density shadows in the surrounding area (white arrow), and there was also pancreatic pseudocyst formation (white stars). These results suggest that patients with SAP have more severe outcome. (TIF) [file pone.0111058.s001.tif]

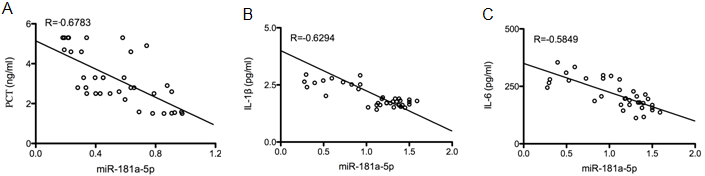

Supplement: Figure S2 — The correlation between the miR-181a-5p and inflammatory cytokines in HTSAP. The correlations between miR-181a-5p and inflammatory cytokines (A) PCT, (B) IL-1β and (C) IL-6 were studies, X axis represents miR-181a-5p, and Y axis represents the levels of inflammatory cytokines. The correlations were analysed by Pearson correlation. “R” represents correlation coefficient. “-” represents negative correlation. (TIF) [file pone.0111058.s002.tif]
