# Supplementary material for: From Moderately Severe to Severe Hypertriglyceridemia Induced Acute Pancreatitis: Circulating MiRNAs Play Role as Potential Biomarkers
Source: PLoS One. 2014 Nov 3;9(11):e111058. doi: 10.1371/journal.pone.0111058 (PMC4218837; doi:10.1371/journal.pone.0111058)
Supplement: Table S1 — Clinical characters of HTAP. The table shows the relevant clinical data of HTAP patients. It can be seen that, compared with the MSAP group, in the SAP group the levels of the biochemical markers TG, TC, Ca, FG, ALT, and AST, as well as the levels of the inflammation-related factors IL-6, IL-1β, and PCT changed significant (P<0.05), whereas age and ALB did not differ significantly between the two groups. The amylase, lipase and trypsinogen-II were elevated obviously in both MSAP and SAP groups, and the level of Try-II in SAP group was much higher than in MSAP groups (P<0.05). Among total 36 SAP patients, there are 20 patients with heart injury, 25 patients with lung injury and 8 patients with kidney injury over 48 hours, conversely no patients have organ failure over 48 hours in MSAP group. The mortality rate of SAP is 11%, which is higher than MSAP (0). All data were presented with mean ±SEM; differences between MSAP and SAP means with P values greater than 0.05 were regarded as statistically significant. (DOC) [file pone.0111058.s003.doc]

Table S1. Clinical characters of HTAP

| MSAP SAP P-Value Reference value  (n=43) (n=36) | | | | |
| --- | --- | --- | --- | --- |
| Gender Male  Female | 19  24 | 15  21 |  |  |
| Age(year) | 40.74±1.397 | 42.28 ±1.534 | 0.9702 |  |
| TG(mmol/l) | 12.85±0.9410 | 24.47 ±2.312 | <0.0001 | 0.4-1.65 |
| TC (mmol/l) | 2.904 ±0.7083 | 13.38 ±1.007 | 0.0304 | 2.9-5.98 |
| Ca++ (mmol/l) | 1.8880±0.0847 | 1.475±0.1141 | 0.0129 | 2-2.8 |
| FBG (mmol/l) | 8.223 ±0.550 | 18.71 ±1.124 | 0.0001 | 3.9-6.1 |
| Serum AMY (IU/L) | 759.3 ±231.8 | 911.7 ±315.1 | 0.0612 | 28-100 |
| LPS (IU/L) | 763.1 ±197.5 | 886.3 ±212.3 | 0.0557 | 0-60 |
| Try-Ⅱ(ng/ml) | 83.77±15.21 | 135.58±19.26 | 0.0001 | 0.9±0.12 |
| ALT(IU/l) | 57.2 ±12.65 | 68.60 ±16.22 | 0.0326 | 0-64 |
| AST (IU/l) | 69.40 ±18.38 | 97.38 ±23.35 | 0.0300 | 0-42 |
| ALB (g/l) | 33.62 ±1.355 | 31.64 ±2.335 | 0.7427 | 35-53 |
| IL-6(pg/ml) | 120.08±10.67 | 210.6±12.89 | 0.0385 | 27.19±12.56 |
| IL-1β(pg/ml) | 0.7622 ±0.1559 | 1.7280 ±0.2648 | 0.0050 | 0.2617±0.045 |
| PCT(ng/ml) | 1.32±0.12 | 3.85±1.41 | 0.0002 | <0.05 |
| Organ failure（>48h）Heart  Lung  Kidney | 0  0  0 | 20  25  8 |  |  |
| Mortality rate (%) | 0(0/43) | 11 (4/36) |  |  |

*All data were presented with Mean ± SEM, differences between MSAP and SAP means with P values <0.05 were regarded as being statistically significant.
