# Supplementary material for: From Moderately Severe to Severe Hypertriglyceridemia Induced Acute Pancreatitis: Circulating MiRNAs Play Role as Potential Biomarkers
Source: PLoS One. 2014 Nov 3;9(11):e111058. doi: 10.1371/journal.pone.0111058 (PMC4218837; doi:10.1371/journal.pone.0111058)
Supplement: Table S2 — GOs and involved genes targeted by overlap miRNAs. The false discovery rate (FDR) was calculated to correct the P value. Enrichment degree means the contribution of miRNAs to the GOs. The key functions in the network always have the higher enrichment degrees (P<0.01). (DOC) [file pone.0111058.s004.doc]

Table S2. GOs and involved genes targeted by overlap miRNAs

| **go_name** | **pvalue** | **FDR** | **enrichment** | **gene_symbol** |
| --- | --- | --- | --- | --- |
| fibroblast growth factor receptor signaling pathway | 4.38E-15 | 2.07E-13 | 7.355633803 | CALM1, SOS1, PTEN, ERBB3, PRKCD, MAP2K1, KRAS, NRG1, MAPK1, CREB1, KIT, FOXO1, ADCY2, FGF7, ADRBK1, KITLG, TNRC6B, PHLPP2, CDKN1B, PIK3R1, TRIM71, PDGFRA, IRS2, GSK3B, KIF16B, PRKCE |
| apoptotic process | 6.16E-14 | 2.585E-12 | 3.441626825 | KALRN, PAWR, PRKCB, TNFRSF11B, KPNB1, PDCD6IP, GREM1, PTEN, BNIP3L, PAX3, PHLDA1, ARHGEF7, DBNL, PIM1, UNC5B, BCL2L1, TP63, STK24, TNF, ARHGEF3, FOXO1, SUDS3, EPHA3, SOS1, PRKCD, TGFBR1, KPNA1, APAF1, RPS6KB1, YWHAG, BCL2L11, RNF34, ZMAT3, HMGB2, FGD4, CFLAR, TNFAIP1, LUC7L3, DCC, GATA6, SEMA6A, ZFAND6, ITSN1, BIRC6, BMF, MAPK1, IL1A, C6orf120, ARF6, RAD21, PPP3R1 |
| negative regulation of apoptotic process | 7.999E-14 | 3.24E-12 | 3.920978349 | TCF7L2, BNIP3L, CFLAR, CDKN1B, BIRC6, RPS6KB1, VHL, IL2, FOXO1, TNF, UBE2B, PIM1, BCL2L1, NRG1, CBX4, DDX3X, STRADB, TSC22D3, TFAP2B, CCNG1, PRLR, GATA6, IGF1, HNF1B, WT1, NOTCH2, TGFBR1, TAF9B, ATM, WNK3, IL1A, PIK3R1, PTEN, KDR, HSP90B1, TP63, VEGFA, BAG4, SERPINE1, GSK3B, CYR61, SIRT1, WNT7A |
| intracellular signal transduction | 7.672E-12 | 2.609E-10 | 4.662021424 | PRKCD, WSB2, SMAD2, KALRN, ADCY2, GPR155, ARHGEF3, PLCL2, GRIP1, PAG1, SHC3, SOCS4, DEPTOR, PRKCB, GSK3B, CIT, RAPGEF2, RGS6, AKAP7, SH2B3, DCLK1, RASA1, CBLB, CDC42BPA, ARHGEF7, TNS3, GUCY1B3, PRKCE, PRKCH, ITSN1 |
| cellular lipid metabolic process | 3.742E-10 | 9.224E-09 | 5.964027408 | TBL1XR1, PPARA, SLC25A1, GRHL1, CYP7A1, TBL1X, AGPAT9, ACSL1, CHD9, NFYB, ELOVL7, GPD2, ACSL6, GLIPR1, LCLAT1, GPD1L, NCOA6, NCOA2, AGPS, SP1 |
| innate immune response | 1.31E-09 | 2.856E-08 | 3.106892256 | POLR3D, PCBP2, DDX3X, ABI1, FGF7, TNRC6B, ADCY2, KITLG, KRAS, CREB1, NRG1, HSP90AA1, PRKCE, SRPK2, ERBB3, CDKN1B, CALM1, HSP90B1, POLR3G, KIT, FOS, FOXO1, PRKCD, IRS2, PIK3R1, CD4, SOS1, OTUD5, SAMHD1, GSK3B, ADRBK1, ADARB1, BCL2L1, PTEN, MAP2K1, PDGFRA, MAPK1, ZC3HAV1, PHLPP2 |
| gene expression | 2.728E-09 | 5.728E-08 | 2.840948385 | RNMT, NR1D2, POLR3G, MED8, IGF2BP2, ZFP36L1, CNOT2, RNGTT, CSTF1, NR4A3, PAIP1, PCBP2, THRB, NUDT21, SRSF7, RORA, PPP2R2A, SP1, EIF4A2, SSR1, PRKCD, NR6A1, PPARA, XRN1, LSM11, ADARB1, EDA, EIF3J, NR3C1, ESR1, NOTCH2, NR2C2, SMAD2, POLR3D, TNPO1, PGR, SERPINE1, POU2F1, CNOT6, SNRPD1, E2F5, HNRNPA3, TNRC6B |
| response to DNA damage stimulus | 5.635E-09 | 1.123E-07 | 5.409950023 | UBE2B, RNF169, H2AFX, NCOA6, FBXO45, SESN1, DYRK2, MCTS1, TIMELESS, SIRT1, CCNK, ATM, FOXO1, MAPK1, TAOK1, NIPBL, RNF8, ZMAT3, ZBTB40 |
| induction of apoptosis | 1.474E-08 | 2.569E-07 | 4.849868441 | DCC, PLAG1, MAPK1, PRKCD, BCL2L11, PAWR, NOTCH2, CDKN1B, PTEN, BNIP3L, PHLDA1, PLAGL2, ACVR1B, PRKCE, ZMAT3, TNF, WT1, TGFBR1, NAIF1, TP63 |
| phospholipid metabolic process | 3.46E-08 | 5.399E-07 | 5.125215811 | GPCPD1, AGPAT9, MBOAT1, PIK3C2A, MBOAT2, INPP4A, LCLAT1, GPD1L, MTMR14, GATA6, PI4K2B, PISD, LPCAT2, PITPNB, PTEN, PIK3R1, INPP5E, PIK3R3 |
| negative regulation of cell proliferation | 3.638E-08 | 5.627E-07 | 3.451805807 | B4GALT1, NOTCH2, CLMN, TNF, WT1, PAWR, ADARB1, SSTR1, PKP2, ABI1, PTHLH, TFAP2B, IGF1, VHL, CDKN1B, MAP2K1, TOB1, E2F7, STRN, CTBP2, NRK, AXIN2, PTEN, RBBP4, IL1A, SESN1, SMAD2, ADM |
| cell proliferation | 3.706E-08 | 5.713E-07 | 3.546466298 | RBBP7, NR6A1, PRDM4, TCF7L2, BCL2L1, DDIT4, INSIG1, IRS2, PTEN, EHF, MAP2K1, KITLG, PDXK, AXIN2, IL1A, COPS2, RAP1B, PAX3, VEGFA, CDV3, IRF2, ZFP36L2, PDAP1, ZMYND11, PIM1, CYR61, NRG1 |
| cell migration | 1.776E-07 | 2.5E-06 | 5.563084389 | CUL3, NRG1, TNFAIP1, USP33, TNS3, VEGFA, LIMD1, EPHA3, RPS6KB1, CDH2, CDC42BPA, PTEN, FLT1, GSK3B, PEAK1 |
| wound healing | 3.414E-06 | 3.584E-05 | 6.89590669 | DCBLD2, C6orf89, DCN, PDGFRA, MIA3, TGFBR1, LOX, ERBB3, NRG1, PPARA |
| negative regulation of fat cell differentiation | 9.817E-06 | 8.762E-05 | 9.96569741 | INSIG1, SIRT1, TCF7L2, ENPP1, ZFPM2, FOXO1, TNF |
| negative regulation of insulin receptor signaling pathway | 1.081E-05 | 9.431E-05 | 12.60965795 | ENPP1, GRB10, KANK1, RPS6KB1, PRKCD, PRKCB |
| insulin receptor signaling pathway | 1.525E-05 | 0.0001272 | 4.174819185 | STRADB, GRB10, FOXO1, FGF7, PIK3C2A, PIK3R1, IRS2, PIK3R3, SOS1, RPS6KB1, MAP2K1, SHC3, KRAS, MAPK1 |
| cytokinesis | 2.391E-05 | 0.0001857 | 5.586557319 | RAD21, NEK7, CHMP1B, BCL2L1, PDS5A, CUL3, CIT, RAB11A, NIPBL, RASA1 |
| negative regulation of cell growth | 2.975E-05 | 0.0002211 | 4.526543879 | WT1, NAIF1, ACVR1B, DDX3X, RBBP7, CDKN1B, ENPP1, GREM1, DCBLD2, FOXK1, SIRT1, CAPRIN2 |
| negative regulation of glycogen biosynthetic process | 3.411E-05 | 0.0002456 | 22.06690141 | GSK3B, ENPP1, GRB10, GFPT1 |
| fat cell differentiation | 3.63E-05 | 0.0002573 | 6.922949461 | TCF7L2, ARID5B, TFAP2B, OSBPL8, TBL1X, FOXO1, NIPBL, GSK3B |
| intrinsic apoptotic signaling pathway | 6.408E-05 | 0.0004207 | 6.419462228 | BCL2L1, BCL2L11, CUL3, PPP3R1, BMF, DDX3X, YWHAG, APAF1 |
| response to glucose stimulus | 9.503E-05 | 0.0005894 | 6.087421078 | PRKCD, SMAD2, IRS2, ACVR1C, TCF7L2, RPS6KB1, HNF1B, ACVR2B |
| response to steroid hormone stimulus | 0.0002243 | 0.0011552 | 14.71126761 | ACSL6, LOX, SPP1, HMGB2 |
| cytokine-mediated signaling pathway | 0.0002734 | 0.0013367 | 3.064847418 | KPNA3, EIF4A2, POM121, IL1A, CAMK2B, KPNA4, PRKCD, NUP153, KPNA1, KLF6, KRAS, IRF2, KPNB1, CAMK2D, KIT |
| cellular calcium ion homeostasis | 0.000333 | 0.0015457 | 5.116962645 | CACNB4, PRKCB, TMEM165, STC2, VAPB, ATP2A2, STC1, RYR3 |
| positive regulation of apoptotic process | 0.0003554 | 0.0016185 | 3.136412383 | ATM, TNF, BCL2L1, DDX3X, BCL2L11, PAWR, PIK3R1, ARHGEF7, CYR61, SIRT1, TCF7L2, SUDS3, CNR1, BNIP3L |
| glycerophospholipid biosynthetic process | 0.0003822 | 0.0017026 | 4.462968824 | MBOAT1, LCLAT1, PISD, GPD1L, MBOAT2, PITPNB, GPCPD1, LPCAT2, AGPAT9 |
| cellular response to insulin stimulus | 0.0005297 | 0.0021456 | 5.516725352 | TBC1D4, IRS2, ACSL6, GFPT1, UBE2B, PRKCD, ENPP1 |
| positive regulation of glucose import | 0.0006306 | 0.0024578 | 8.172926448 | PRKCD, IRS2, CREBL2, IGF1, PIK3R1 |
| response to mechanical stimulus | 0.0006969 | 0.0026505 | 6.304828974 | PTGER4, RPS6KB1, TNF, FOS, PRKCD, DCN |
| cellular response to hypoxia | 0.0008522 | 0.0030672 | 4.469245855 | PRKCE, STC1, SIRT1, ARNT, GATA6, VHL, VEGFA, STC2 |
| transforming growth factor beta receptor signaling pathway | 0.0008962 | 0.0031772 | 3.64742172 | SP1, FOS, NLK, SERPINE1, E2F5, TGFBRAP1, SMAD2, FKBP1A, TGFBR1, USP9Y |
| apoptotic signaling pathway | 0.0009175 | 0.0032293 | 3.972042254 | ARHGEF7, ARHGEF3, SOS1, TNF, ITSN1, FGD4, KALRN, SENP1, BCL2L11 |
| long-chain fatty-acyl-CoA biosynthetic process | 0.0009853 | 0.0033902 | 10.38442419 | ELOVL7, ACSL6, ACSL1, SLC25A1 |
