# Supplementary material for: From Moderately Severe to Severe Hypertriglyceridemia Induced Acute Pancreatitis: Circulating MiRNAs Play Role as Potential Biomarkers
Source: PLoS One. 2014 Nov 3;9(11):e111058. doi: 10.1371/journal.pone.0111058 (PMC4218837; doi:10.1371/journal.pone.0111058)
Supplement: Table S3 — Pathways and genes targeted by overlap miRNAs. The false discovery rate (FDR) was calculated to correct the P value. Enrichment degree means the contribution of miRNAs to the signal pathways. Key signal pathways of the network always have higher enrichment degrees (P<0.01). (DOC) [file pone.0111058.s005.doc]

Table S3. Pathways and involved genes targeted by overlap miRNAs

| **pathway_name** | **pvalue** | **FDR** | **enrichment** | **gene_name** |
| --- | --- | --- | --- | --- |
| PI3K-Akt signaling pathway | 9.47009E-14 | 6.01788E-12 | 4.578723059 | SOS1, DDIT4, FGF11, FGF7, PDGFRA, HSP90B1, PIK3R3, YWHAG, PTEN, FLT1, PPP2R5E, CREB1, BCL2L1, MAP2K1, IGF1, IL2, HSP90AA1, GNB4, PRLR, CDKN1B, VEGFA, ITGB8, RPS6KB1, SPP1, GSK3B, BCL2L11, KDR, MAPK1, KITLG, KRAS, PPP2R2A, KIT, AKT3, COL6A6, PIK3R1, PHLPP2 |
|  |  |  |  |  |
| HIF-1 signaling pathway | 4.7089E-09 | 2.30179E-08 | 6.661706086 | PIK3R3, PRKCB, FLT1, VHL, ARNT, MAP2K1, IGF1, CAMK2D, SERPINE1, CDKN1B, VEGFA, RPS6KB1, CAMK2B, MAPK1, AKT3, PIK3R1 |
| Insulin signaling pathway | 4.39226E-08 | 1.36019E-07 | 5.359104628 | AKT3, KRAS, GSK3B, SOS1, MAPK1, SOCS4, PPP1R3B, PIK3R3, FOXO1, SHC3, PTPRF, RPS6KB1, CBLB, MAP2K1, CALM1, IRS2, PIK3R1 |
| T cell receptor signaling pathway | 4.73375E-08 | 1.43244E-07 | 6.129694836 | AKT3, MAPK1, CD4, PPP3R1, SOS1, GSK3B, TNF, FOS, PIK3R3, NFAT5, IL2, MAP2K1, CBLB, PIK3R1, KRAS |
| TGF-beta signaling pathway | 6.95234E-08 | 1.98832E-07 | 7.083202921 | DCN, TGFBR1, SP1, SMAD2, ZFYVE16, ACVR1C, RPS6KB1, E2F5, ACVR2A, ACVR1B, ACVR2B, MAPK1, TNF |
| Focal adhesion | 1.20106E-07 | 3.05292E-07 | 4.284835225 | SOS1, PDGFRA, RAP1B, GSK3B, PRKCB, PTEN, PIK3R3, FLT1, IGF1, MAP2K1, SHC3, VEGFA, ITGB8, SPP1, PIK3R1, KDR, PPP1R12B, COL6A6, MAPK1, AKT3 |
| B cell receptor signaling pathway | 2.81007E-07 | 6.60682E-07 | 6.968495182 | AKT3, MAPK1, PPP3R1, SOS1, GSK3B, PRKCB, FOS, PIK3R3, NFAT5, MAP2K1, PIK3R1, KRAS |
| MAPK signaling pathway | 3.08986E-07 | 7.17107E-07 | 3.7343987 | PDGFRA, FGF11, PRKCB, FGF7, RAP1B, TGFBR1, FOS, MAP3K3, NLK, MAP2K1, RASA1, CACNB4, MAPK1, IL1A, NTF3, AKT3, KRAS, TAOK1, RAPGEF2, TNF, PPP3R1, SOS1 |
| Wnt signaling pathway | 3.58124E-07 | 8.12764E-07 | 4.938047868 | GSK3B, PRKCB, TCF7L2, NLK, NFAT5, FZD3, WNT7A, AXIN2, VANGL1, CAMK2D, CTBP2, PRICKLE2, CAMK2B, TBL1XR1, TBL1X, PPP3R1 |
| VEGF signaling pathway | 6.17862E-07 | 1.30876E-06 | 7.245848224 | PPP3R1, PIK3R3, PRKCB, NFAT5, MAP2K1, VEGFA, KDR, MAPK1, AKT3, KRAS, PIK3R1 |
| GnRH signaling pathway | 2.35967E-06 | 4.15128E-06 | 5.756582976 | KRAS, SOS1, GNRHR, MAPK1, MAP3K3, ADCY2, PRKCB, CAMK2D, MAP2K1, CALM1, CAMK2B, PRKCD |
| Fc gamma R-mediated phagocytosis | 2.97988E-06 | 5.11784E-06 | 5.634102487 | AKT3, MAPK1, PRKCB, PIK3R3, ARF6, MARCKSL1, MAP2K1, RPS6KB1, PRKCE, PIK3R1, WASF3, PRKCD |
| Pancreatic cancer | 4.5643E-06 | 7.40685E-06 | 6.686939821 | MAPK1, PIK3R1, TGFBR1, AKT3, KRAS, BCL2L1, SMAD2, VEGFA, PIK3R3, MAP2K1 |
| p53 signaling pathway | 6.03849E-06 | 9.35909E-06 | 6.49026512 | ZMAT3, CCNG1, SESN1, PTEN, CCNG2, RCHY1, IGF1, SERPINE1, APAF1, ATM |
| Gastric acid secretion | 1.49256E-05 | 2.07125E-05 | 5.884507042 | ATP1B1, GNAI3, KCNK2, ADCY2, KCNK10, PRKCB, ATP1B2, CAMK2D, CALM1, CAMK2B |
| mRNA surveillance pathway | 1.5458E-05 | 2.13177E-05 | 5.276867728 | PPP2R5E, NUDT21, MAGOHB, RNMT, PAPOLG, DAZAP1, CSTF1, CPSF6, HBS1L, PPP2R2A, RNGTT |
| Long-term depression | 1.59385E-05 | 2.18574E-05 | 6.620070423 | GNAI3, KRAS, GRIA3, MAPK1, PRKG1, PRKCB, IGF1, MAP2K1, GUCY1B3 |
| Chemokine signaling pathway | 1.79483E-05 | 2.40513E-05 | 3.677816901 | PRKCB, RAP1B, PIK3R3, ADCY2, ADRBK1, MAP2K1, GNB4, SHC3, GSK3B, MAPK1, AKT3, GNAI3, KRAS, PRKCD, PIK3R1, SOS1 |
| Calcium signaling pathway | 4.12234E-05 | 4.99282E-05 | 3.617524821 | SLC25A4, PDGFRA, PRKCB, ADCY2, ERBB3, RYR3, ATP2B1, ORAI2, ATP2B2, CAMK2D, GRM5, CALM1, CAMK2B, ATP2A2, PPP3R1 |
| Apoptosis | 6.2379E-05 | 7.07848E-05 | 5.015204866 | PIK3R3, BCL2L1, APAF1, IL1A, CFLAR, AKT3, ATM, PPP3R1, TNF, PIK3R1 |
| Pancreatic secretion | 0.000132512 | 0.000135269 | 4.597271127 | ATP1B1, RAP1B, ATP2B1, ADCY2, ATP2B2, RAB11A, RAB27B, PRKCB, ATP1B2, ATP2A2 |
| Endocytosis | 0.000144487 | 0.00014574 | 3.24513256 | PDGFRA, CHMP1B, TGFBR1, FLT1, ERBB3, PDCD6IP, ADRBK1, ARF6, RAB11A, SMAD2, ZFYVE16, CBLB, KDR, KIT, PSD3 |
| Vascular smooth muscle contraction | 0.000188184 | 0.000186603 | 4.045598592 | PRKCB, ADCY2, PRKG1, MAP2K1, CALM1, GUCY1B3, PRKCH, PRKCE, MAPK1, PPP1R12B, PRKCD |
| Endocrine and other factor-regulated calcium reabsorption | 0.000225046 | 0.000220012 | 6.304828974 | CALB1, ATP2B1, RAB11A, ATP1B2, PRKCB, ESR1, ATP1B1 |
| Metabolic pathways | 0.000727589 | 0.000658779 | 1.707447376 | PNPO, LCLAT1, INPP5E, PI4K2B, ST6GALNAC6, PAFAH1B2, PIK3C2A, POLR3G, INPP5A, CPOX, PGAP1, PDXK, GFPT1, ACSL1, ENPP1, MBOAT1, ST8SIA1, GNE, MBOAT2, MGAT4A, ACSL6, B4GALT1, MTHFR, CYP7A1, MCCC2, NDST3, MMAB, POLR3D, AGPAT9, GLS, ST6GAL2, LPCAT2, INPP4A, ADO, HMBS, B3GALT5, AGPS, GALNT4, FUT9, ACADSB, COQ2, ST3GAL5, CHSY1, PANK3, POC1B-GALNT4, PISD |
| Natural killer cell mediated cytotoxicity | 0.000731273 | 0.000661756 | 3.467655936 | MAPK1, PPP3R1, SOS1, TNF, PRKCB, PIK3R3, NFAT5, MAP2K1, SHC3, PIK3R1, KRAS |
| Cytokine-cytokine receptor interaction | 0.000882174 | 0.000781001 | 2.644722266 | PDGFRA, TGFBR1, FLT1, EDA, IL2, PRLR, VEGFA, ACVR2A, ACVR1B, KDR, ACVR2B, IL1A, KITLG, KIT, TNFRSF11B, TNF |
| Inositol phosphate metabolism | 0.000903685 | 0.000797579 | 5.064534749 | INPP5E, PI4K2B, PTEN, PIK3C2A, INPP5A, IPMK, INPP4A |
