# Supplementary material for: From Moderately Severe to Severe Hypertriglyceridemia Induced Acute Pancreatitis: Circulating MiRNAs Play Role as Potential Biomarkers
Source: PLoS One. 2014 Nov 3;9(11):e111058. doi: 10.1371/journal.pone.0111058 (PMC4218837; doi:10.1371/journal.pone.0111058)
Supplement: Table S4 — Overlap miRNAs targeting gens involved in fat, glucose, insulin, calcium metabolize. Based on the data above, the targeted genes related to pancreas metabolism were showed. (DOC) [file pone.0111058.s006.doc]

Table S4. The overlap miRNAs targeted to the genes involved in fat, insulin, glucose, calcium metabolize

|  | miR-181a-5p | miR-24-3p | miR-222-3p | miR-361-5p | miR1246 |
| --- | --- | --- | --- | --- | --- |
| fat | TBL1XR1, PPARA, TBL1X, ACSL1, CHD9, GPD2, LCLAT1, and GPK1L, SIRT1, ENPP1, TNF, PIK3C2A, INPP4A, LCLAT1, GPD1L, GATA6, PI4K2B, LPCAT2, PITPNB, INPP5E, PIK3R3, TCF7L2, OSBPL8, TBL1X, ACSL1, SIRT1, TCF7L2, ENPP1, ZFPM2 and TNF | AGPAT9, AGPS, SP1, ARID5B and ACSL6 | CYP7A1, NFYB, PIK3R1, ZFPM2, and NIPBL | ELOVL7, GLIPR1, AGPS, SP1 and TFAP2B | GRHL1,CYP7A1, NIPBL, GSK3B |
| insulin | ENPP1, GRB10, KANK1, RPS6KB1, PRKCD, PIK3C2A, IRS2, PIK3R3, SOS1, MAP2K1, SHC3, KRAS, MAPK1, TBC1D4, IRS2, GFPT1, UBE2B, PRKCD | AGPAT9, MTMR14, INSIG1, STRADB, ACSL6 | PIK3R1 | PRKCB, FGF7 |  |
| glucose | GRB10, GFPT1, PRKCD, IRS2, TCF7L2, RPS6KB1, ACVR2B, PRKCD, IRS2, GRB1, GFPT1, LCLAT1, GPD1L, MBOAT2, PITPNB, GPCPD1, LPCAT2, TBC1D4, GFPT1, UBE2B, and ENPP1 | ACVR1C, HNF1B, AGPAT9 | PIK3R1 |  | GSK3B, CREBL2 |
| calcium | TMEM165, ATP2A2, STC1 and RYR3 |  | CACNB4 ,VAPB | PRKCB, VAP |  |
